# The International Bathymetric Chart of the Arctic Ocean Version 4.0

**DOI:** 10.1038/s41597-020-0520-9

**Published:** 2020-07-09

**Authors:** Martin Jakobsson, Larry A. Mayer, Caroline Bringensparr, Carlos F. Castro, Rezwan Mohammad, Paul Johnson, Tomer Ketter, Daniela Accettella, David Amblas, Lu An, Jan Erik Arndt, Miquel Canals, José Luis Casamor, Nolwenn Chauché, Bernard Coakley, Seth Danielson, Maurizio Demarte, Mary-Lynn Dickson, Boris Dorschel, Julian A. Dowdeswell, Simon Dreutter, Alice C. Fremand, Dana Gallant, John K. Hall, Laura Hehemann, Hanne Hodnesdal, Jongkuk Hong, Roberta Ivaldi, Emily Kane, Ingo Klaucke, Diana W. Krawczyk, Yngve Kristoffersen, Boele R. Kuipers, Romain Millan, Giuseppe Masetti, Mathieu Morlighem, Riko Noormets, Megan M. Prescott, Michele Rebesco, Eric Rignot, Igor Semiletov, Alex J. Tate, Paola Travaglini, Isabella Velicogna, Pauline Weatherall, Wilhelm Weinrebe, Joshua K. Willis, Michael Wood, Yulia Zarayskaya, Tao Zhang, Mark Zimmermann, Karl B. Zinglersen

**Affiliations:** 1 Department of Geological Sciences, Stockholm University, Stockholm, Sweden; 2 Bolin Centre for Climate Research, Stockholm University, 106 91 Stockholm, Sweden; 3 Center for Coastal and Ocean Mapping, University of New Hampshire, Durham, NH USA; 4 National Institute of Oceanography and Applied Geophysics, Sgonico, Italy; 5 CRG Marine Geosciences, Department of Earth and Ocean Dynamics, University of Barcelona, Barcelona, Spain; 6 Department of Earth System Science, University of California, Irvine, CA USA; 7 Alfred-Wegener-Institut, Helmholtz Centre for Polar and Marine Research, Bremerhaven, Germany; 8 Access Arctic, Le Vieux Marigny, France; 9 Geophysical Institute, University of Alaska, Fairbanks, AK USA; 10 College of Fisheries and Ocean Sciences, University of Alaska, Fairbanks, AK USA; 11 Italian Hydrographic Service, Genoa, Italy; 12 Geological Survey of Canada, Dartmouth, Nova Scotia Canada; 13 Scott Polar Research Institute, University of Cambridge, Cambridge, UK; 14 UK Polar Data Centre, British Antarctic Survey, Cambridge, UK; 15 Canadian Hydrographic Service, Burlington, Ontario Canada; 16 Geological Survey of Israel, Jerusalem, Israel; 17 Norwegian Mapping Authority, Hydrographic Service, Stavanger, Norway; 18 Korea Polar Research Institute, Incheon, South Korea; 19 GEOMAR Helmholtz Centre for Ocean Research Kiel, Kiel, Germany; 20 Greenland Institute of Natural Resources, Nuuk, Greenland; 21 Geological Survey of Denmark and Greenland, Copenhagen, Denmark; 22 Department of Earth Science, University of Bergen, Bergen, Norway; 23 Institut des Geosciences de l’Environment, Universite Grenoble Alpes, CNRS, Grenoble, France; 24 Danish Geodata Agency, Danish Hydrographic Office, Ålborg, Denmark; 25 University Centre in Svalbard, Svalbard, Norway; 26 Lynker Technologies, Seattle, USA; 27 Jet Propulsion Laboratory, California Institute of Technology, Pasadena, CA USA; 28 Tomsk Polytechnic University, Tomsk, Russia; 29 Laboratory of Arctic Studies, V.I. Il’ichov Pacific Oceanological Institute, Far Eastern Branch of the Russian Academy of Sciences, Vladivostok, Russia; 30 Canadian Hydrographic Service, Darthmouth, Nova Scotia Canada; 31 British Oceanographic Data Centre, Liverpool, UK; 32 Geological Institute, Russian Academy of Sciences, Moscow, Russian Federation; 33 Second Institute of Oceanography, Ministry of Natural Resources, Beijing, China; 34 NOAA National Marine Fisheries Service, Alaska Fisheries Science Center, Seattle, USA

**Keywords:** Geomorphology, Geophysics, Ocean sciences

## Abstract

Bathymetry (seafloor depth), is a critical parameter providing the geospatial context for a multitude of marine scientific studies. Since 1997, the International Bathymetric Chart of the Arctic Ocean (IBCAO) has been the authoritative source of bathymetry for the Arctic Ocean. IBCAO has merged its efforts with the Nippon Foundation-GEBCO-Seabed 2030 Project, with the goal of mapping all of the oceans by 2030. Here we present the latest version (IBCAO Ver. 4.0), with more than twice the resolution (200 × 200 m versus 500 × 500 m) and with individual depth soundings constraining three times more area of the Arctic Ocean (∼19.8% versus 6.7%), than the previous IBCAO Ver. 3.0 released in 2012. Modern multibeam bathymetry comprises ∼14.3% in Ver. 4.0 compared to ∼5.4% in Ver. 3.0. Thus, the new IBCAO Ver. 4.0 has substantially more seafloor morphological information that offers new insights into a range of submarine features and processes; for example, the improved portrayal of Greenland fjords better serves predictive modelling of the fate of the Greenland Ice Sheet.

## Abstract (summary)

**Table:** 

Measurement(s)	depth
Technology Type(s)	digital curation
Factor Type(s)	geographic location
Sample Characteristic - Environment	ocean floor
Sample Characteristic - Location	Arctic Ocean

Machine-accessible metadata file describing the reported data: 10.6084/m9.figshare.12369314

## Background & Summary

A broad range of Arctic climate and environmental research, including questions on the declining cryosphere and the geological history of the Arctic Basin, require knowledge of the depth and shape of the seafloor^1–3^. Bathymetry provides the geospatial framework for these and other studies^4^ and has impact on many processes, including the pathways of ocean currents and, thus, the distribution of heat^5,6^, sea-ice decline^7^, the effect of inflowing warm waters on tidewater glaciers^8^, and the stability of marine-based ice streams and outlet glaciers grounded on the seabed^9–11^. Bathymetric data from large parts of the Arctic Ocean are, however, not available or extremely sparse due to difficulties, both logistical and political, in accessing the region^12^.

The International Bathymetric Chart of the Arctic Ocean (IBCAO) project, was initiated in 1997 in St Petersburg, Russia, to address the need for up-to-date digital portrayals of the Arctic Ocean seafloor^13^. Since 1997, three Digital Bathymetric Models (DBMs) have ingested new data sets compiled by the IBCAO project team and have been released for public use^14–16^. These DBMs comprised grids with a regular cell size of 2.5 × 2.5 km (Ver. 1.0), 2 × 2 km (Ver. 2.0) and 500 × 500 m (Ver. 3.0) on a Polar Stereographic projection. Depth estimates for grid cells between constraining depth observations were interpolated by the continuous curvature spline in a tension gridding algorithm^17^. All depth data available at the time of the compilations were used, including multi- and single-beam bathymetry, and contours and soundings digitized from depth charts, with direct depth observations having the highest priority and digitized contours the lowest^18^.

Recognizing the importance of complete global bathymetry, the General Bathymetric Chart of the Ocean (GEBCO), a project under the auspices of the International Hydrographic Organization (IHO) and the Intergovernmental Oceanographic Commission (IOC), teamed up with the Nippon Foundation of Japan and jointly launched the Seabed 2030 project in 2018 with the goal of mapping all of the world ocean by 2030^19^. The first release from the Seabed 2030 project was the *GEBCO_2019* global grid, with a grid-cell size of 15 × 15 arc seconds^20^. The Arctic Ocean is poorly represented by this geographical grid because the grid cells are greatly distorted in the longitudinal direction at high latitudes. Seabed 2030 is built on the IBCAO model; a focused effort to gather and assemble all available bathymetric data into a digital database that is then used to compile a DBM. Seabed 2030 has established four Regional Centers, one of which (shared by Stockholm University and the University of New Hampshire) has responsibility for the Arctic Ocean. With the establishment of Seabed 2030, the IBCAO has merged its efforts with Seabed 2030 and, while keeping its well-established identity, the compilation of updated versions of IBCAO will now be conducted under the auspices of the Seabed 2030 Arctic Regional Center.

**Fig. 1:**
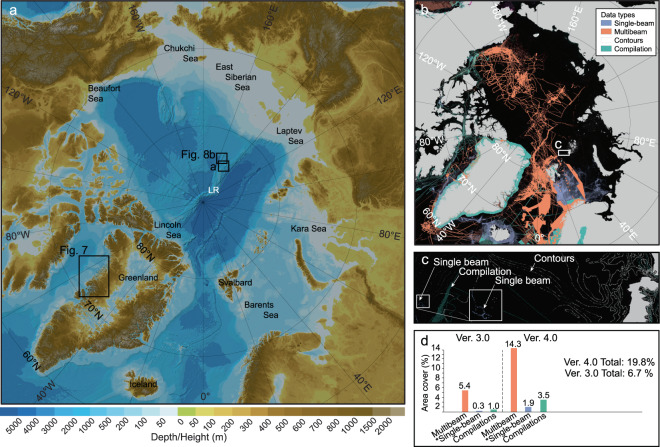
**(a)** Shaded relief map of IBCAO Ver. 4.0 with the under-ice topography of Greenland from BedMachine Ver. 3 shown. **(b)** Map of Ver. 4.0 data sources grouped into the data types (TID) listed in Table 1. **(c)** Close-up showing an area with single-beam soundings and digitized depth contours used in gridding. Since these data types occupy relatively few grid cells, they are difficult to see in the overview map shown in **(b)**. **(d)** Summary statistics of the proportion of the IBCAO area covered by the different data types in Ver. 4.0 and 3.0. The data types “steering points” and “interpolated depths” are not shown in **(a)** as they are not counted as part of the depth data (Methods; Table 1). *Refers to “Isolated soundings”, “ENC soundings” and “Mixture of direct measurement methods”, which are merged with data type “Single-beam” sounding on the map as well as in the summary statistics shown in (**d**). LR: Lomonosov Ridge.

Here we present IBCAO Ver. 4.0, incorporating new data sources and compiled using an improved gridding algorithm and with a finer grid-cell size of 200 × 200 m on a Polar Stereographic Projection. Recognizing that the lateral resolution achievable by a surface-ship deployed echo-sounder varies as a function of depth (decreasing resolution with depth), the Seabed 2030 project has defined target grid-cell sizes that are also variable by depth^19^ (see Methods Section). The data coverage within the Ver. 4.0 area is therefore calculated with respect to the Seabed 2030 target resolutions. In total, ∼19.8% of the gridded area is constrained by some form of bathymetric data, excluding digitized bathymetric contours, whereas the comparable coverage for IBCAO Ver. 3.0 was calculated as ∼6.7% (Fig. 1) using the variable resolution grid. Ver. 4.0 has ∼14.3% of the gridded area comprised of modern multibeam echo-sounder derived bathymetry whereas Ver. 3.0 had ∼5.4%. This implies that the new Ver. 4.0 has ∼2.7 times the area of the Arctic Ocean constrained by multibeam bathymetry relative to Ver. 3.0. One of the important additions to IBCAO Ver. 4.0 is the recently released IceBridge BedMachine Ver. 3 topography/bathymetry grid of Greenland^21^, containing both Greenland ice-surface and under-ice topography, yielding a seamless transition to the adjacent seafloor along most of the margins of the Greenland Ice Sheet, which is critical for ice-sheet modelling and for improving projections of the impact of Greenland on future sea level rise. The IBCAO DBM will be updated continuously as new data become available.

## Methods

### Grid compilation

**Fig. 2:**
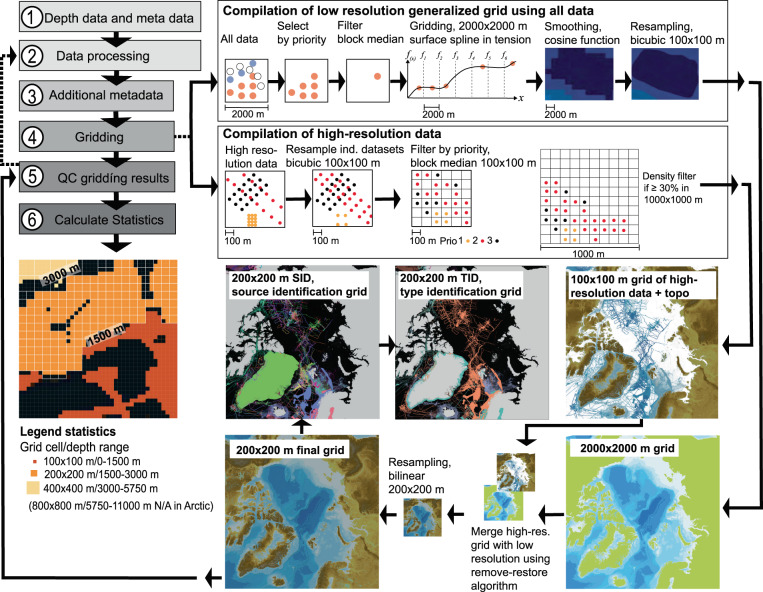
Schematic illustration of the IBCAO DBM compilation work flow.

The IBCAO DBM compilation workflow, illustrated schematically in Fig. 2, contains six main steps. Step 1 consists of assembling the different kinds of contributed depth data listed in Table 1 along with necessary metadata. The metadata follow the standard adopted by EMODnet Bathymetry^22^, with the additions shown in Online-only Table 1. Contributions to IBCAO come in various forms. Ideally, contributions are cleaned bathymetric data in the form of XYZ points representing spot soundings, single-beam soundings, nodes of high-resolution multibeam grids, or nodes of digitized contours from bathymetric maps. Gridded compilations derived from multiple sources have also been contributed (see sub-section ‘Source data’ and Online-only Table 2; the latter only available online) as well as raw multibeam bathymetry requiring processing. All gathered XYZ datasets are reviewed using QPS Qimera software. If necessary, additional post-processing is applied in Step 2 using tools available in Qimera including, for example, removal of outliers or adjustments of vertical levels where systematic offsets are evident. If datasets of relatively poor quality are found to be in conflict with other observations, they may be completely or partially removed. In Step 3, additional metadata are included; most importantly the version number of each dataset is incremented if it has been modified, permitting roll-back through the processing history.

**Table 1:** The source data used in the IBCAO Ver. 4.0 compilation classified into data types (TID; Type Identification). In the calculated statistics of mapped area, types 13, 14 and 17 are included in type 10 whereas 41 and 72 are counted as no data.

TID	Data type	Description
10	Singlebeam	Depth value collected by a singlebeam echo-sounder
11	Multibeam	Depth value from grid derived from multibeam echo-soundings
17	Combination of direct measurement methods	Depth values from single beam, spot sounding or a combination of other direct measurements. Crowd sourced bathymetry from, for example Olex, falls under this category
41	Interpolated based on a computer algorithm	Depth value is an interpolated value based on a computer algorithm (e.g. spline in tension). These are counted as no data in statistics describing coverage
42	Digital bathymetric contours from charts	Depth values taken from digitized bathymetric contours
70	Pre-generated grid	Depth value is taken from a pre-generated grid that in turn is based on mixed source data types (e.g. single beam, multibeam, interpolation etc.)
72	Steering points	Depth value used to constrain the grid in areas of poor data coverage. These are counted as no data in statistics describing coverage
13	Isolated sounding	Depth value that is not part of a regular ship survey or trackline, (e.g. spot soundings through sea ice)
14	ENC sounding	Depth value extracted from an Electronic Navigation Chart (ENC)

In Step 4, the processed XYZ data are gridded using a modified version of the algorithm applied to compile IBCAO Ver. 3.0^15^. First, a low-resolution grid with a cell-spacing of 2000 × 2000 m is produced. The depth data passed forward are selected based on their quality prioritization within each 2000 × 2000 m grid cell. Multibeam data are generally prioritized before single-beam and spot-sounding data which, in turn, are prioritized ahead of digitized depth contours from charts. A block median filter is then applied using the Generic Mapping Tools (GMT)^23^. The block median filtered data are subsequently gridded using the GMT routine *surface*, which applies a continuous curvature spline in tension function^17^. The tension parameter is set to 0.34. This value was decided on after analyses of the gridding results over the course of the IBCAO-project. A value of 0 implies no tension of the spline surface, whereas a tension of 1 removes the curvature altogether by not permitting maxima or minima between constraining data points. The resulting 2000 × 2000 m grid is smoothed using a cosine filter over 6000 m in GMT to provide a smooth base over which higher-resolution data are merged. The smoothed grid is then resampled to 100 × 100 m.

Higher resolution datasets (i.e. multibeam surveys and some gridded compilations) are individually down-sampled (if high enough in resolution) to 100 × 100 m. If multiple contrasting depths exist for one grid cell, the depths passed forward to the block median filter at 100 × 100 m are selected based on the same prioritization as used for the 2000 × 2000 m grid cells. The final step in the preparation of the high-resolution data consists of a density filter, which only passes forward data if more than 30% of an area of 1000 × 1000 m is covered by depth values.

The final action within Step 4 consists of merging the high-resolution data passed forward from the procedure described above with the 100 × 100 m resampled 2000 × 2000 m smoothed grid by applying a remove-and-restore approach^24^. This involves the calculation of the difference between the 2000 × 2000 m grid resampled to 100 × 100 m and the high-resolution 100 × 100 m datasets remaining after applying the density filter. The differences, or residuals, are then gridded using the surface spline in tension function before they are added back onto the low-resolution 2000 × 2000 m grid (resampled to 100 × 100 m). This procedure results in a smooth merging of the high-resolution data onto the low-resolution resampled grid. To prevent introducing spline-function artifacts, the residuals are forced to be zero at a distance of 1000 m from the data. Finally, the entire grid is resampled to 200 × 200 m. The gridding algorithm is written in Python, from which the applied GMT routines are called.

Step 5 consists of a quality check of the final grid using a Stockholm University developed web interface along with Qimera and the Open Source Geographic Information System QGIS, version 3.8.3-Zanzibar, which has also been used to produce the maps displayed in this data description^25^. The web interface has a mark-up function permitting all members in the IBCAO Regional Mapping Committee to take part in the quality control. If issues are found and marked, the associated source data are passed back to Step 2 for further analysis and processing. Step 6 in Fig. 2 is described in the following sub-section.

### Calculation of statistics

Echo sounders mounted on surface vessels increase their ensonified area with increasing depth, thus decreasing their achievable mapping resolution with depth. Based on this principle, Seabed 2030 defined a set of target mapping resolutions: 0–1500 m, 100 × 100 m; 1500–3000 m, 200 × 200 m; 3000–5750 m, 400 × 400 m; and 5750–11000 m, 800 × 800 m^19^. Since IBCAO contributes to the Seabed 2030 project, the data coverage calculated in Step 6 uses the Seabed 2030 resolutions. For example, a depth sounding between 3000–5750 m is considered to map an area of 400 × 400 m whereas a sounding with a value between 0–1500 m only maps an area of 100 × 100 m. Where the source data are available in the form of multibeam, single-beam and spot soundings, it is thus relatively easy to calculate how much of the IBCAO grid is mapped or not. However, when the contributed data are compilation grids, the estimated surveyed area is uncertain as we do not know the underlying data coverage. Even if only the nodes of the contributed grids at their native resolution (i.e. before resampling) are counted, they will likely overestimate the mapped area. For this reason, gridded compilations are kept as a separate category (Fig. 1).

## Data Records

### Source data

**Fig. 3:**
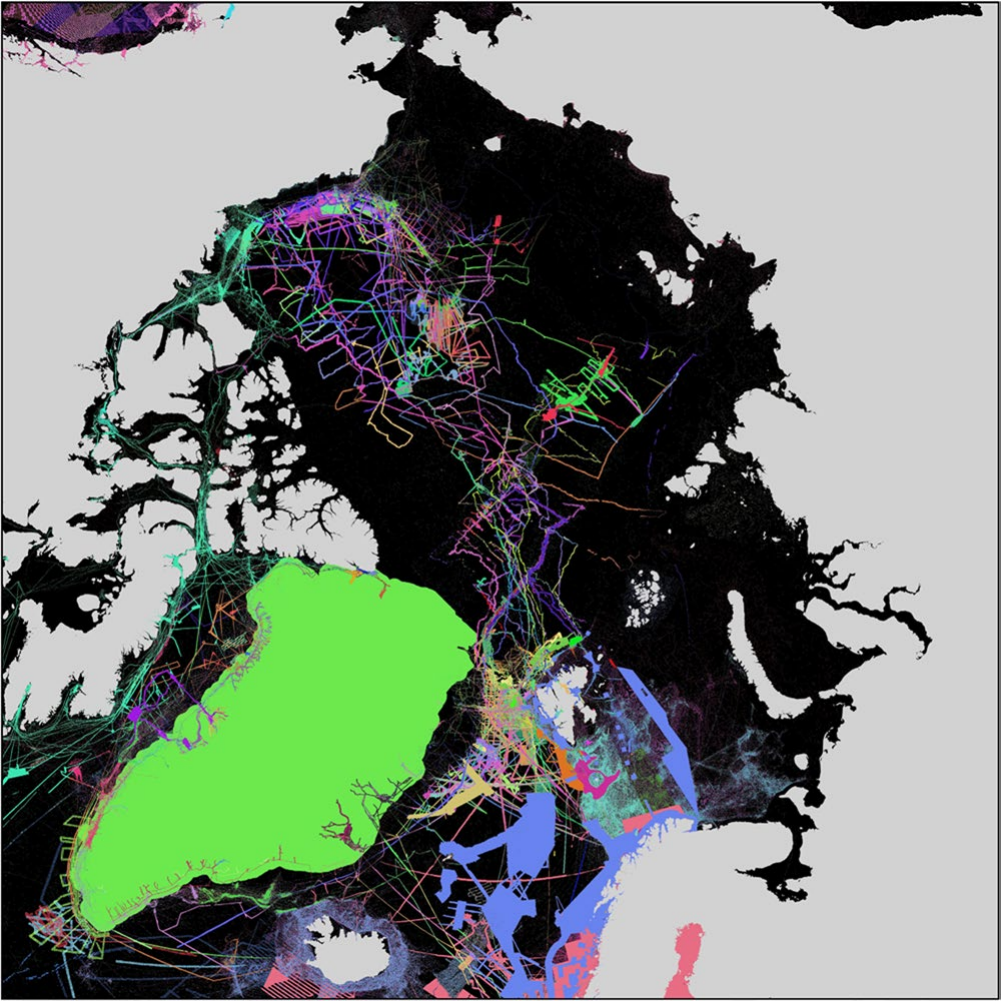
Map showing the underlying sources for IBCAO Ver. 4 based on the Source Identification grid (SID) available for download. The source of the depth used within a specific 200 × 200 m grid-cell in the gridding is linked by a unique number to a database record containing the source metadata. Legend is not included as there are 505 SIDs.

**Fig. 4:**
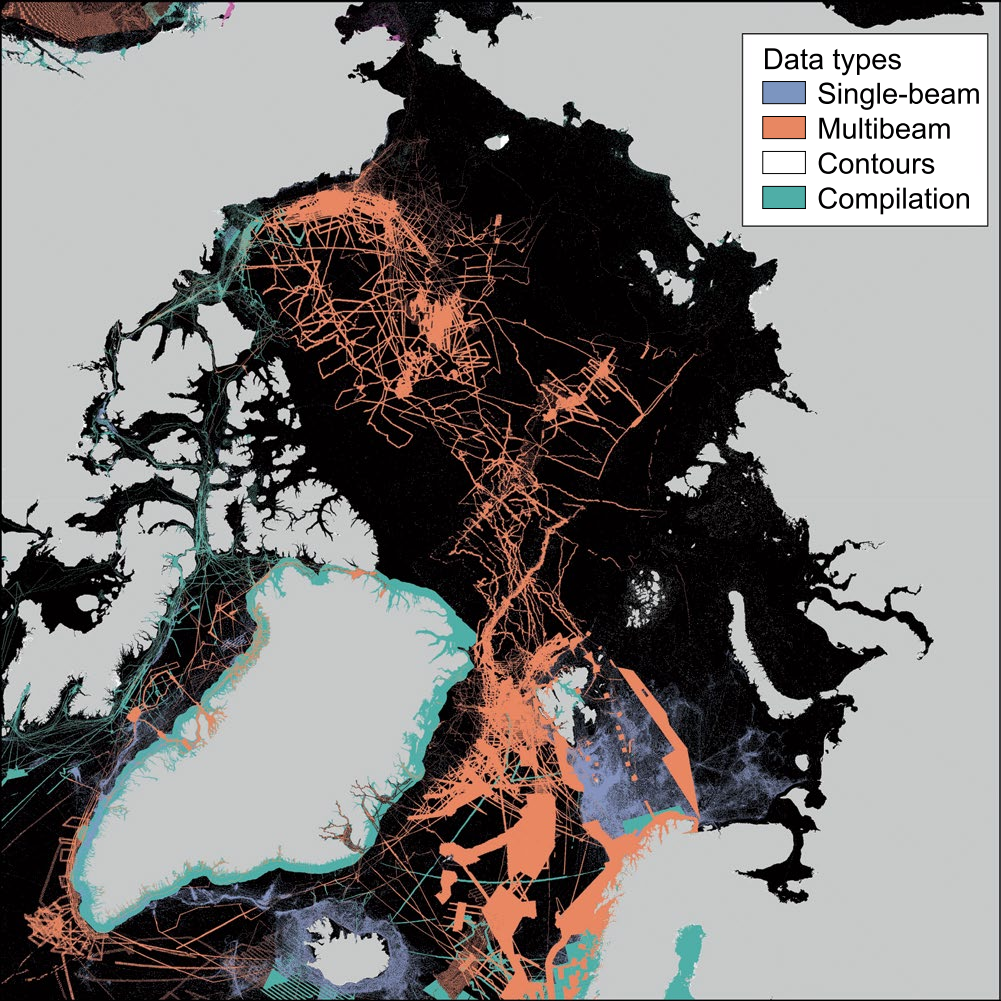
Map showing the underlying sources for IBCAO Ver. 4 classified into the data types listed in Table 1. “Isolated soundings”, “ENC soundings” and “Combination of direct measurement methods” listed in Table 1 are merged with data type “Single-beam” in this map. Note that contours and single-beam soundings hardly show at this scale.

The IBCAO Ver. 4 is available for download from the British Oceanographic Data Centre^26^. The bathymetric source data for IBCAO Ver. 4 are listed in Online-only Table 2 along with references where available. Individual surveys are, in most cases, aggregated to one contributing organization. Each dataset is assigned a Source Identification number (SID) and Type Identification number (TID). The former links each dataset to its full metadata whereas the latter groups the data into the categories listed in Table 1. SID and TID grids are compiled within the workflow in Fig. 2 (See SID and TID maps in Figs. 3 and 4). Spatially, the largest contributed gridded compilations are BedMachine Ver. 3 covering the coastal waters of Greenland^21^, MAREANO mapping a significant portion of the Norwegian EEZ, EMODnet encompassing European Arctic waters, including the part of Bay of Bothnia covered by IBCAO^22^, and NONNA-100 composed of bathymetric data from Canadian waters released by the Canadian Hydrographic Service at a resolution of approximately 100 m. BedMachine Ver. 3 also provides the under-ice topography of Greenland at a gridded horizontal resolution of 150 m, derived from ice-thickness measurements from NASA’s Operation IceBridge and other surveys using ice-penetrating radar and an ice-mass conservation algorithm in the coastal areas^21^. The bathymetry in BedMachine Ver. 3 is, for the most part, linked back to IBCAO Ver. 3.0, RTopo-2^27^ and the DBM by Arndt, *et al*.^28^ of northeastern Greenland, apart from within the fjords where a kriging algorithm is used to interpolate depths between the under-ice topography and available bathymetric data, including recent surveys along the Greenland coastline carried out by the NASA Earth Venture Suborbital mission named Oceans Melting Greenland^8,29^. We have masked BedMachine Ver. 3 so it is used from the outer coast of Greenland, resulting in a vastly improved fjord representation compared with other bathymetric models. Bathymetric data from Greenland coastal waters gathered since BedMachine Ver. 3 have been merged using the remove-and-restore approach. These include, for example, multibeam surveys of Petermann and Sherard Osborn fjords in northwest Greenland^30^ and additional bathymetry collected and compiled within NASA’s Ocean Melting Greenland^31,32^.

The area covered by “crowd sourced” bathymetry has increased substantially in Ver. 4.0 compared to Ver. 3.0 through contributions from fishing vessels and other ships using Olex (www.olex.no) and MaxSea (http://www.maxsea.com/) mapping systems, the latter in Greenland waters only. Since 2012, when IBCAO Ver. 3.0 was compiled, numerous icebreaker expeditions mapping the seafloor with multibeam sonar in the sea-ice covered Arctic Ocean have been completed. These include expeditions with Canadian CCGS *Amundsen* and CCGS *Louis S. St-Laurent*, German RV *Polarstern*, Swedish icebreaker *Oden*, and USCGC *Healy* (Online-only Table 2).

## Technical Validation

### Validation: Comparison between IBCAO Vers. 3.0 and 4.0

**Fig. 5:**
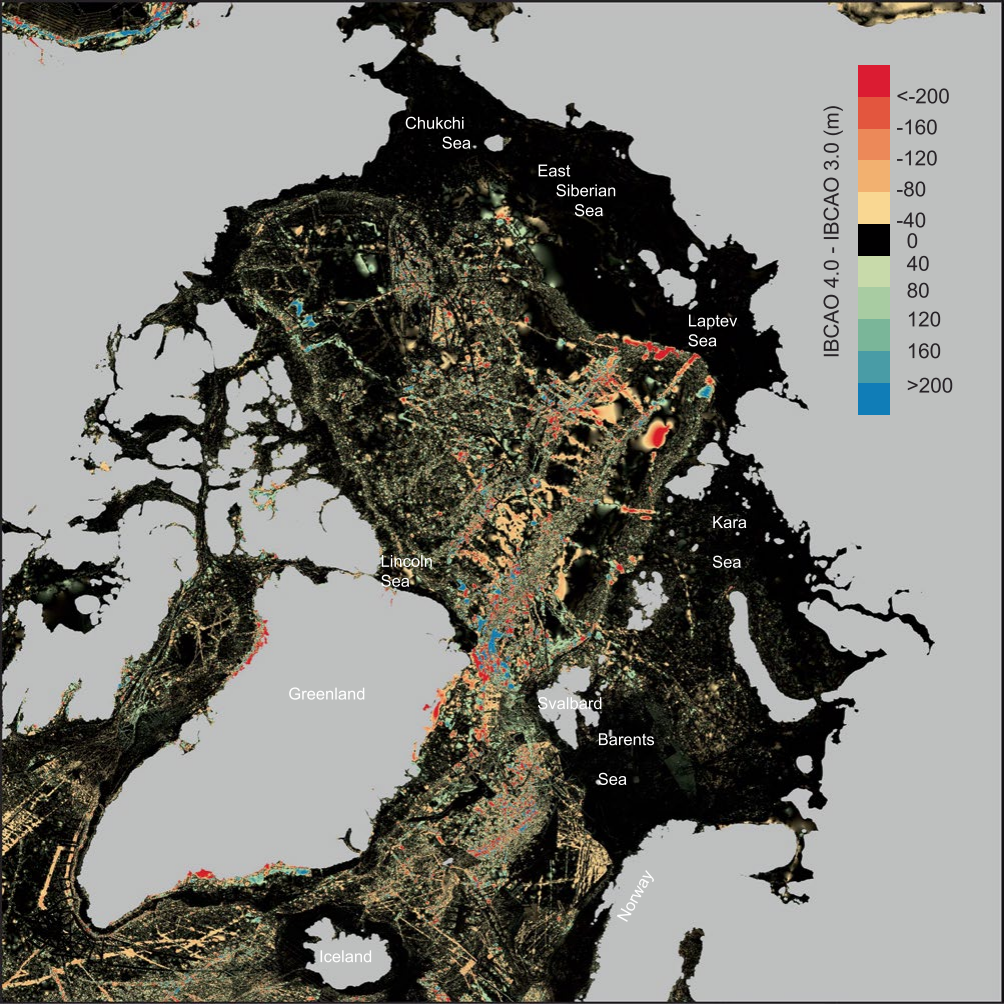
Map showing the difference in meters between IBCAO Ver. 3.0 and 4.0, generated by subtracting Ver. 3.0 from 4.0. Positive values imply shallower depths in IBCAO Ver. 3.0 and vice versa.

**Fig. 6:**
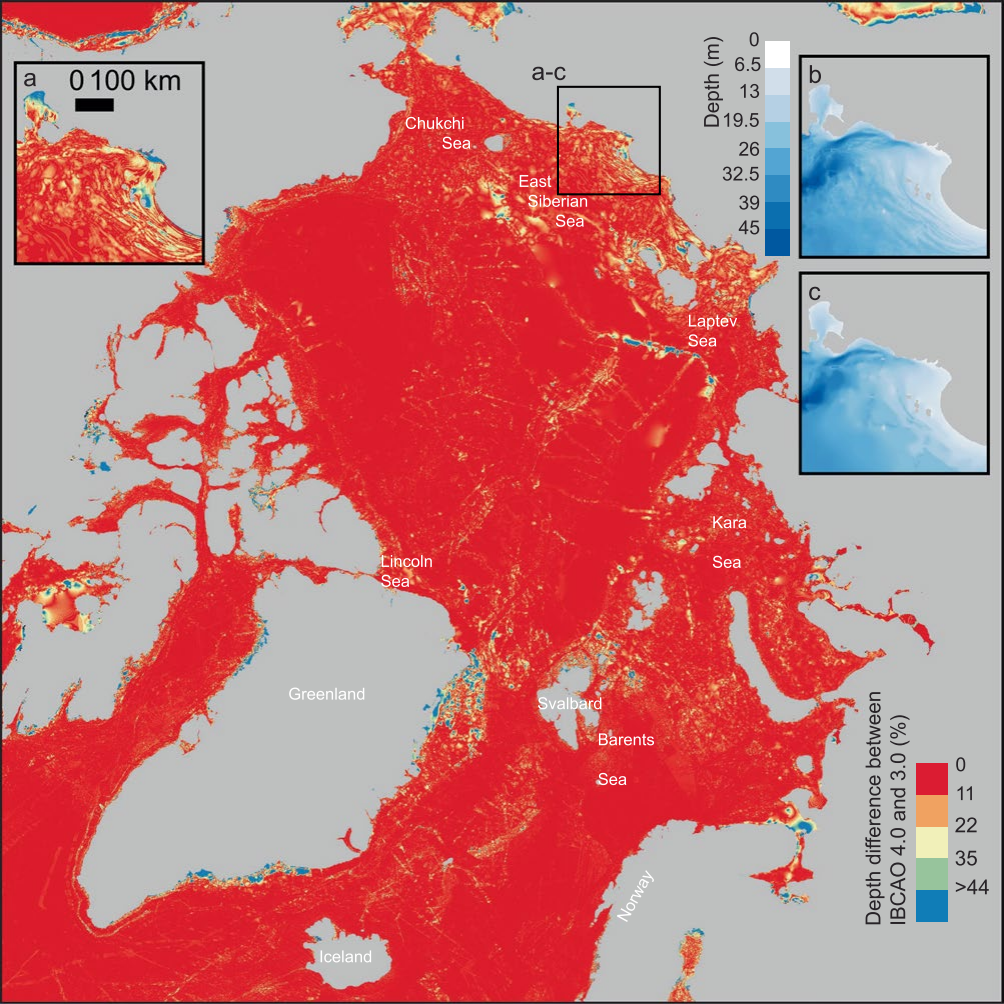
Map showing the depth difference in percent between IBCAO Ver. 3.0 and 4.0 (i.e. the absolute depth difference between Ver. 4.0 and 3.0 divided by the absolute depth of Ver. 4.0). This reveals the updates in the shallow areas of the grid (i.e. mainly the large continental shelf areas). (**a**) Zoom-in on an area in the East Siberian Sea showing that substantially more details are distinguishable in IBCAO Ver. 4.0 (shown in **b**) compared to Ver. 3.0 (shown in **c**).

The improvements in IBCAO Ver. 4.0 compared to earlier versions result from the large amount of new bathymetric data including gridded compilations, an improved gridding algorithm, and a higher resolution. This is best illustrated by specific examples, together with an overview map showing the depth differences between IBCAO Vers. 3.0 and 4.0, generated by subtracting Ver. 3.0 from 4.0, that highlights the most significantly updated areas (Fig. 5). The new multibeam bathymetry is readily visible in the difference map as well as in the improved representation of fjords along sections of the Greenland coast (Fig. 5). In general, the least updated areas in terms of absolute depth changes are located on the Russian continental shelf, in the Barents Sea between southern Svalbard and northern Norway, and on the Norwegian and Iceland continental shelves (Fig. 5). The lack of updates in Russian waters stems from the fact that no new multibeam data has been contributed from these areas, despite their collection during Russian efforts to map the extent of their juridical continental shelf. If we look at the updates as a function of how much the depth has changed relative to water depth (i.e. the percent depth change), the East Siberian and Laptev seas show some clear differences in Ver. 4.0 compared to 3.0 (Fig. 6). The updates result from the fact that individual soundings on charts were used, rather than digitized contours from charts, providing more bathymetric detail (Fig. 6). These soundings were digitized by Danielson, *et al*.^33^ for the purpose of compiling the Alaska Region Digital Elevation Model (ARDEM). Areas that do not show large depth differences were already relatively well mapped in IBCAO Ver. 3.0. If the Barents Sea is examined carefully, the new additions from the MAREANO compilation are clearly visible (Fig. 5).

**Fig. 7:**
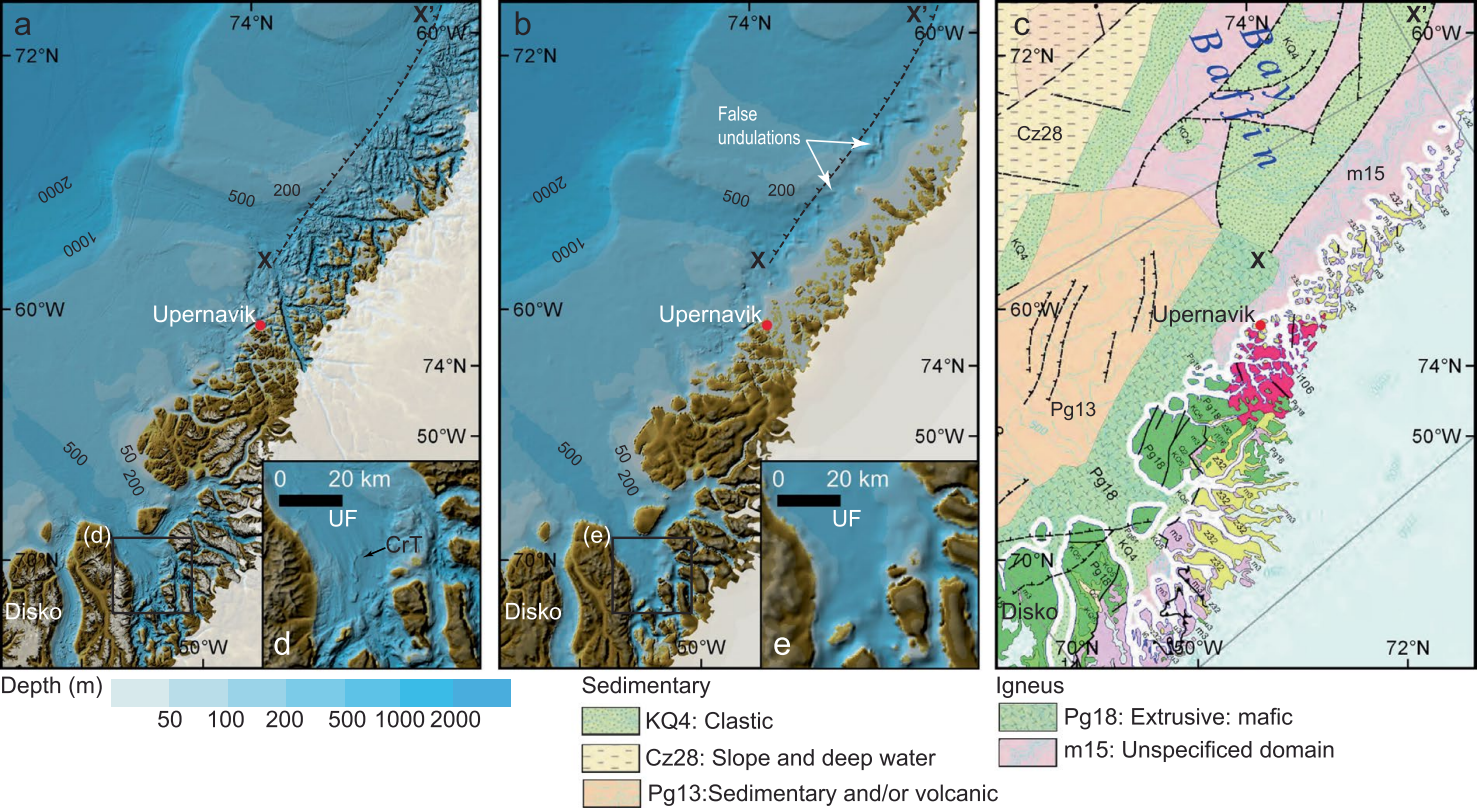
Comparison off western Greenland between IBCAO Ver. 4.0 **(a)**, Ver. 3.0 **(b)** and the geological map by Harrison, *et al*.^34^ **(c)**. The thrust fault marked X-X’ on the geological map is shown as a reference on the bathymetric maps in **(a,b)**. The seafloor morphology changes markedly across the marked thrust fault in Ver. 4.0. The inset **(d)** shows how subglacial landforms in the form of Crag-and-Tails (CrT) are visible in Ver. 4, whereas they are not in Ver. 3.0 **(e)**. UF: Uummannaq Fjord. See location in Fig. 1.

The incorporation of BedMachine Ver. 3 and additional merging of all bathymetry available since its release not only enhances the representation of Greenland fjords, but also highlights the complex coastal bathymetry (Fig. 7). This is particularly noticeable off the western coast of Greenland between about 55°N and 75°N, where IBCAO Ver. 4.0 reveals a rough submarine landscape characterized by criss-crossing channels that commonly occur where the seafloor is composed of igneous bedrock (Fig. 7). The transition to a smoother seafloor morphology on the outer continental shelf occurs rather abruptly across a near straight southwest-to-northeast trending line that fits well with geological maps showing change across a thrust fault from igneous rocks to a seafloor composed of sedimentary rocks further offshore^34^ (Fig. 7).

Lack of depth data from the western Greenland inner continental shelf in IBCAO Ver. 3.0 resulted in a poorly constrained spline function causing undulations that do not represent the “true” seafloor morphology in this area (Fig. 7b). The Uummannaq Fjord of western Greenland is a good example, showing that submarine glacial landforms with spatial dimensions on the order of hundreds of meters, such as glacially streamlined drumlins and large mega-scale glacial lineations images using multibeam, are distinguishable in the IBCAO Ver. 4.0 DBM (Fig. 7d). This can only be the case when the gridding is based on high-resolution bathymetry, here collected by RRS *James Clark Ross*^35^.

**Fig. 8:**
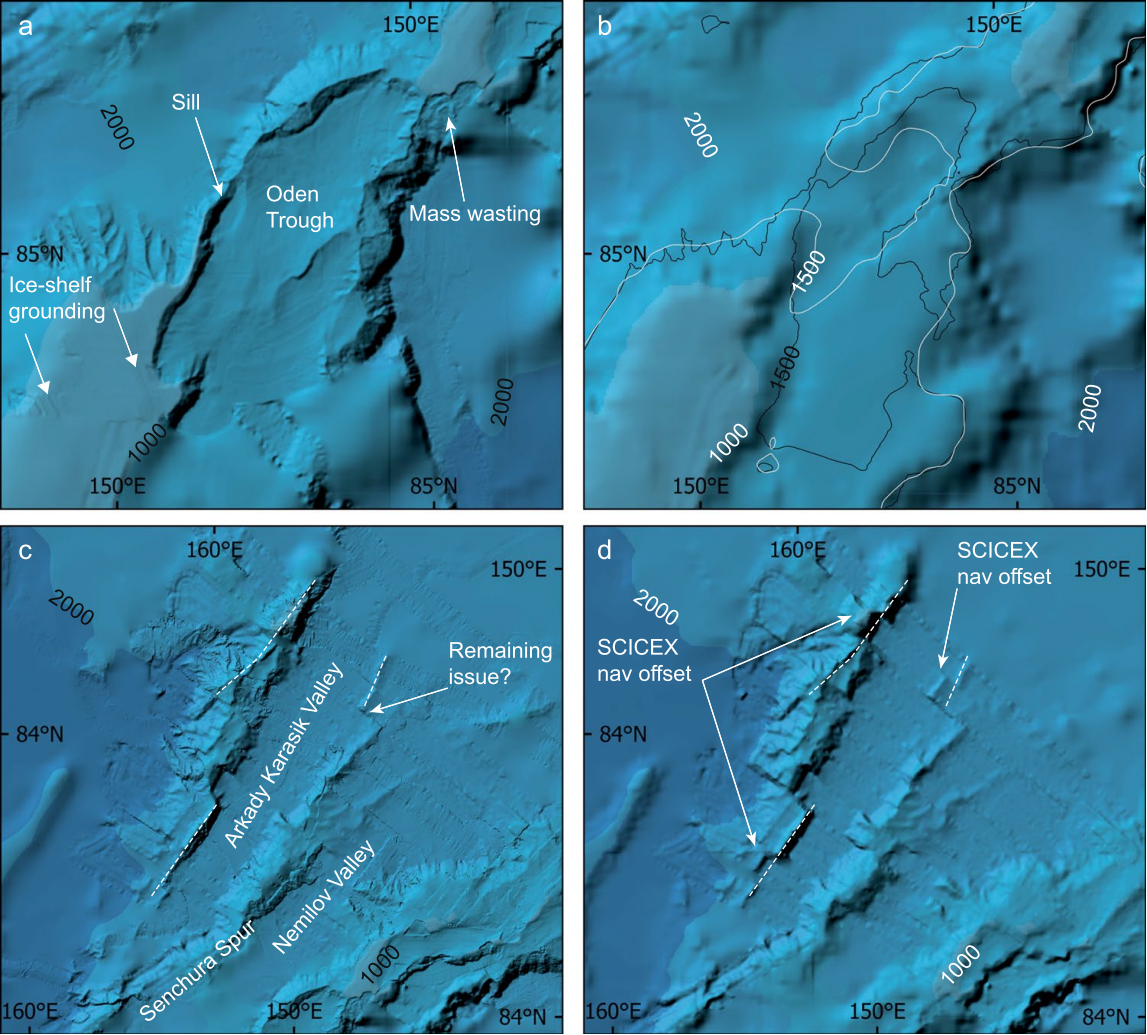
Comparison between IBCAO Ver. 3.0 and Ver. 4.0 in two areas of the Lomonosov Ridge (Fig. 1). (**a**) Systematic multibeam surveys in 2014 by Swedish icebreaker *Oden* mapped a trough formed in the ridge crest, *Oden Trough*, and a critical sill depth influencing water exchange across the ridge^6^. In addition, lineations were mapped on the ridge crest, interpreted to be formed by a grounded ice shelf during the penultimate glaciation at about 140 000 years ago^37^. None of these features could be seen in IBCAO Ver. 3.0 (**b**) because it was compiled in this area through gridding of bathymetric contours retrieved from the Russian map “Bottom relief of the Arctic Ocean”^43^. The 1500 m isobaths derived from Ver. 3.0 (white) and 4.0 (black) shown in b clearly illustrate the large bathymetric differences between the two versions in the area of the sill. (**c**) The portrayal of the two spurs extending from the Lomonosov Ridge at about 84°N 155–160°E, one of them named Senchura Spur, are improved in Ver. 4.0 compared to Ver. 3.0 (**d**) due to additional multibeam bathymetry and adjustment of navigational issues in SCICEX 1999 (see main text).

The Lomonosov Ridge extends >1600 km across the central Arctic Ocean between the continental shelves of Northern Greenland and Siberia (Fig. 1). Details of the ridge came to light in the first published version of IBCAO^16^ where it was drastically remapped compared to the GEBCO Sheet 5.17^36^, which had served as the authoritative international bathymetric map of the Arctic Ocean for nearly two decades before the IBCAO project began. Numerous multibeam surveys with icebreakers have been carried out over the Lomonosov Ridge since the release of IBCAO Ver. 3.0, (Online-only Table 2), leading again to a substantially improved bathymetry (Fig. 8). Examples include surveys that have been individually published revealing critical sills that influence water exchange across the Lomonosov Ridge^6^, ice-shelf grounding on the ridge crest^37^, and where the foot of the slope is located along the ridge flanks, identified for the purpose of substantiating Denmark’s submission under Article 76 of the United Nations Convention on the Law of the Sea (UNCLOS)^38^.

The Science Ice Exercise (SCICEX) was a program utilizing US Navy nuclear submarines for systematic mapping under the Arctic Ocean pack ice between 1993 and 2001^39^. Of the eight completed expeditions, two (1998 and 1999) involved acquisition of swath bathymetry using the specifically designed sonar system Seafloor Characterization and Mapping Pod (SCAMP)^39^. This swath bathymetry was used in IBCAO Ver. 3.0, although in many areas newer multibeam bathymetry has now replaced the SCICEX data; for example along the Northern Alaskan margin and on Chukchi Borderland, where several mapping expeditions with USCGC *Healy* have been carried out to collect seafloor bathymetry in support of the establishment of a U.S. extended continental shelf under Article 76 of UNCLOS^40^. A major caveat with SCICEX/SCAMP data has been the problem of precisely geo-registering the swath bathymetry, which is particularly evident where areas have been systematically surveyed and the locations of seafloor features are noticeably offset on different tracks (Fig. 8c,d). To resolve this issue in areas that were based solely on SCICEX/SCAMP bathymetry and appeared to show large ‘fault offsets’, we used multibeam surveys that cross over the SCICEX tracks to re-position the swath data (Fig. 8c,d). These multibeam surveys were positioned using modern GPS implying a User Range Error (URE) commonly not exceeding 10 m. The result is not perfect but is a significant improvement in IBCAO Ver. 4.0 compared to Ver. 3.0.

### Errors

Despite the fact that the IBCAO Ver. 4.0 DBM is a substantial improvement over previous versions, it is certainly not free of errors. The DBM remains limited by its underlying source database. The uncertainties associated with the depths of grid cells depend on a variety of factors including the approach used to correct soundings for sound speed, vertical referencing, navigation, and echo-sounder uncertainties. In addition, the gridding process will affect the final depth assigned to each grid cell. The random error component is thus a difficult parameter to derive, primarily because of lack of metadata on the widely varying data sources and the fact that some contributions are in the form of gridded compilations. In several areas we still rely on digitized contours from published maps for which the underlying source data are unknown. While the random error component of DBMs have been estimated using statistical modeling approaches^41,42^, we do not provide this for IBCAO Ver. 4.0 because the metadata are not sufficient to provide a classification to a large enough portion of the database. Instead, the accompanying TIDs and SIDs provide information that is useful for users when addressing the reliability of IBCAO Ver. 4.0. In addition, we have assembled two grids aimed to further assist users in assessing the reliability of the DBM: minimum and maximum depth grids. These grids report the minimum and maximum depth value for each grid cell, implying a depth range where the block median filter had several input depth values in one grid cell.

## Usage Notes

The most common uses of the IBCAO DBM are map-making and/or geospatial analyses using GIS software and other tools capable of displaying geographic information. The DBM is provided in netCDF and GeoTIFF formats, which are readily imported into most standard GIS software, for example QGIS and ArcMap. The ‘x’ and ‘y’ variables within the netCDF/GeoTIFF grid files represent the grid cell positions, along the x and y axis, in Polar Stereographic projection coordinates (meters), with a true scale set at 75°N. For the DBM, the ‘z’ value represents elevation in meters, depths below the sea surface are negative and heights above the sea surface are positive. The horizontal datum for the dataset is WGS 84 and the vertical datum can be assumed to be Mean Sea Level (however, note that there may be vertical reference issues for older observations, which may be due to chart datum). For the TID grid, the ‘band 1’ value represents the TID code, describing the type of data on which the corresponding cell in the DBM grid is based. A list of TID codes is given in Table 1. The projection parameters are provided in the European Petroleum Survey Group (EPSG) database (https://epsg.io/) as code 3996. This database is used by standard GIS software implying that searching for EPSG 3996, or IBCAO, will provide the correct projection and datum for the IBCAO DBM.

The Polar Stereographic coordinates can be converted to geographic using the GMT command *mapproject* with the following parameters:

mapproject [*input_lonlat*] -R-180/180/0/90 -Js0/90/75/1:1 -C -F > [*output_ xy*]

where *input_lonlat* is a table with longitude and latitude geographic coordinates and *output_xy* is a table with the resulting converted xy Polar Stereographic coordinates. The inverse conversion from xy to geographic coordinates is achieved by adding -I to the command above. See http://gmt.soest.hawaii.edu/doc/latest/mapproject.html for more information.

The GDAL command *gdaltransform* can also be used to convert between the Polar Stereographic and geographic coordinates by calling for the EPSG codes 3996 (IBCAO Polar Stereographic) and 4326 (WGS 84 geographic):

gdaltransform -s_srs EPSG:4326 -t_srs EPSG:3996

The inverse conversion is simply achieved by swapping the order of the EPSG codes. See https://gdal.org/programs/gdaltransform.html for more information.

### Disclaimer information

Version 4.0 of the International Bathymetric Chart of the Arctic Ocean (IBCAO) grid, now referred to as the ‘IBCAO Ver. 4.0 Grid’, is available from https://www.gebco.net/. It is provided on behalf of the IBCAO project under the terms of the disclaimer information as given below.

The IBCAO Ver. 4.0 Grid, should NOT be used for navigation or for any other purpose involving safety at sea. The IBCAO Ver. 4.0 Grid is made available ‘as is’. While every effort has been made to ensure reliability within the limits of present knowledge, the accuracy and completeness of the IBCAO Ver. 4.0 Grid cannot be guaranteed. No responsibility can be accepted by those involved in its creation or publication for any consequential loss, injury or damage arising from its use or for determining the fitness of the IBCAO Ver. 4.0 Grid for any particular use. The IBCAO Ver. 4.0 Grid is based on bathymetric data from many different sources of varying quality and coverage. As the IBCAO Ver. 4.0 Grid is an information product created by interpolation of measured data, the resolution of the IBCAO Ver. 4.0 Grid may be significantly different to that of the resolution of the underlying measured data.

## Data Availability

The gridding and statistical calculation procedures described in the Methods section are based on open source routines, provided within GMT (https://www.generic-mapping-tools.org/) and GDAL (https://gdal.org/), embedded in Python scripts. Codes are available upon request.

## Online-only Tables

**Online-only Table 1:** The adopted metadata fields based on ISO19115 implemented by the European infrastructure SeaDataNet, with listed IBCAO-specific additions (shown as No-Equivalent).

IBCAO metadata	EMODnet metadata equivalent	Definition
lid	Dataset-id	Unique file identification number
file_number	**No-equivalent**	File number (different versions of the same dataset will have different numbers)
name	Dataset-name	Name of dataset
filename	**No-equivalent**	Name of file including extension.
format	Data format	Format of the bathymetric dataset that was contributed (raw, xyz ascii, grid/DTM)
filesize	Data size	Size of file (kB)
version	**No-equivalent**	File version
ibcao_version	**No-equivalent**	First IBCAO version in which dataset was included
in_emodnet_zone	**No-equivalent**	Whether dataset is located inside EMODnet boundary
already_at_emodnet	**No-equivalent**	Whether dataset is previously included in the EMODnet database
sid	**No-equivalent**	Source identification number
tid	**No-equivalent**	Type identification number
in_gridding	**No-equivalent**	Whether dataset is included in latest gridding
weight	**No-equivalent**	Dataset rank in remove restore
restriction	Access constraints	Access constraints (e.g. public, no access)
shape	Measuring area type	Type of object (e.g. point, polygon or surface)
cruise_name	Cruise name	Name of the cruise, expedition or survey
cruise_id	Cruise id	Unique (in IBCAO Database) cruise identification number, four figures
cruise_report	CSR Identifier	Link to cruise report
scientist	**No-equivalent**	For research expeditions, chief scientist/-s
date_start	Start date	Cruise start date
date_end	End date	Cruise end date
date_format	**No-equivalent**	Definition of date format
harbour_from	**No-equivalent**	Harbor where cruise started
harbour_to	**No-equivalent**	Harbor where cruise ended
originator	Originator centre	Originator(s) of the dataset
provider	Holding centre	Holding centre(s) for the dataset
platform_class	Platform class	Type of vessel
station_name	Station name	Name of vessel
station_id	Station id	Ship callsign
instrument	Intrument	Type of acquisition instrument
instrument_specified	**No-equivalent**	Manufacturer and model of acquisition instrument
positioning_type	Instrument	Type of positioning system
positioning_model	**No-equivalent**	Positioning system manufacturer and model
srs	**No-equivalent**	Coordinate reference system
horizontal_geod_datum	Horizontal datum	Horizontal geodetic datum
vertical_datum	Vertical datum	Vertical datum
horizontal_resolution	Horizontal resolution	Resolution in meters at which the data were contributed (may not be full resolution of data)
vertical_resolution	Vertical resolution	Vertical resolution
gridding_resolution	**No-equivalent**	Resolution in gridding
min_depth	Minimum depth	Minimum depth value in the dataset
max_depth	Maximum depth	Maximum depth value in the dataset
area	**No-equivalent**	Area of polygon
length	**No-equivalent**	Length of polygon
coverage	**No-equivalent**	Coverage in square meters
qi_horizontal	QI_Horizontal	Horizontal quality index, based on specified positioning system
qi_vertical	QI_Vertical	Vertical quality index, based on sounding instrument
qi_age	QI_Age	Age quality index, based on age of the dataset in years
qi_purpose	QI_Purpose	Purpose quality index, based on the survey objectives (transit, bathymetric survey etc.)
abstract	Abstract	Short, descriptive text about the dataset
url	**No-equivalent**	Website where data can be downloaded
protocol	Protocol	Type of protocol to be used for downloading data (e.g. http)
access	Data Access Restriction	Data access restrictions, e.g. web data access
database_reference	**No-equivalent**	Database name
comments	**No-equivalent**	General comments on the dataset
updates	**No-equivalent**	Comments on changes between versions
enterer	**No-equivalent**	Name of person adding the metadata and dataset
added to database	**No-equivalent**	Date when file was uploaded to database or updated

**Online-only Table 2:** Major sources used in the compilation of IBCAO Ver. 4.0. Published peer-review articles and cruise reports linked to the data sources are listed where available in our metadata records. Bathymetric data that have been contributed without metadata are not listed, although used in the compilation where no other data are available.

Alaska Fisheries Science Center of the US National Oceanic and Atmospheric Administration’s National Marine Fisheries Service (NOAA Alaskan Fisheries)	Bathymetry data from the Alaska bathymetry compilations for the Aleutian Islands, central and western Gulf of Alaska and Norton Sound: https://www.afsc.noaa.gov/RACE/groundfish/Bathymetry/default.htm Digitized chart soundings, Alaska: Proofed digitized historical chart soundings from “smooth sheets” covering Alaskan waters^44–47^
Alfred Wegener Institute (AWI)	**81 Cruises of Multibeam data in the Atlantic and Indian Ocean region**. **11 Cruises of multibeam data in the South and West Pacific:** https://www.pangaea.de/ https://www.pangaea.de/expeditions/cr.php/Polarstern https://webapp-srv1a.awi.de/eBathy/datasets2.php
National Institute of Oceanography and Applied Geophysics (OGS), Infrastructures Division; Barcelona University (UB), Department of Stratigraphy, Paleontology and Marine Geosciences (now Department of Earth and Ocean Dynamics); University of Bremen, MARUM – Center for Marine Environmental Sciences; University of Tromsø (UiT), The Arctic University of Norway, CAGE, Centre for Arctic Gas Hydrate; Italian Navy, Italian Hydrographic Institute	OGS provided a combined grid of the following datasets Multibeam bathymetry from EGLACOM cruise with RV OGS-Explora in 2008 to the western Barents Sea margin^48^ Multibeam bathymetry from SVAIS cruise with RV Hesperides 2007 to the western Barents Sea margin^49^ Multibeam bathymetry from DEGLABAR cruise with RV OGS-Explora in 2015 to the western Barents Sea margin^50^ Multibeam bathymetry from EDIPO cruise with RV OGS-Explora in 2015 to the western Barents Sea margin^51^ Multibeam bathymetry **by MARUM** from **MSM30 (CORIBAR)** cruise with RV **M.S. Merian** in 2013 to the western Barents Sea margin^52^ Multibeam bathymetry **by University of Tromsø** from **Glacibar** cruise with RV **Jan Mayen** in **2009** to the western Barents Sea margin^53,54^ Multibeam bathymetry **by Italian Hydrographic Institute** from **High North 17** and 18 cruise with RV **Alliance** in **2017 and 2018** to the western Barents Sea margin^55,56^
British Antarctic Survey (BAS), UK NERC Polar Data Centre	**Multibeam data from three cruises of the RRS James Clark Ross:** https://www.bas.ac.uk/ **JR51, 2000, Greenland and Norwegian Seas** ^57,58^ **JR142, 2006, Svalbard** ^57,58^ **JR175, 2009, West Greenland** ^35^ **JR211, 2008, Svalbard** ^59^
Canadian Hydrographic Service (CHS)	**Non-Navigational (NONNA-100) Bathymetric Data** (gridded compilation)**:** All currently validated, digital bathymetric sources acquired by CHS, combined at a resolution of approximately 100 meters. Contains information licensed under the Open Government Licence – Canada. https://open.canada.ca/data/en/dataset/d3881c4c-650d-4070-bf9b-1e00aabf0a1d
Capricorn Greenland Exploration A/S	**Single beam bathymetry from two surveys in 2008 and 2009:** No publications available
ConocoPhillips	**Single beam navigation data from Baffin Bay seismic surveys:** No publications available Navigation data from 2D-seismic surveys for exploration of hydrocarbons in Baffin Bay, West Greenland, in 2012, conducted by Polarcus DMCC for ConocoPhillips. Released to and provided through Greenland Institute of Natural Resources for the purpose of preparation for publication in IBCAO/GEBCO.
Digitized depth contours from bathymetric maps	**Contours digitized from six published maps are used in the IBCAO Ver. 4.0 compilation where no other data are available** ^43,60–63^
EMODnet (gridded compilation)	**The EMODnet Digital Bathymetry (DTM) 2018:** A multilayer bathymetric product for Europe’s sea basins, based upon more than 9400 bathymetric survey data sets and Composite DTMs gathered from 49 data providers from 24 countries^22^
Geological Institute, Russian Academy of Sciences (GIN RAS)	**Multibeam data from four surveys with RV Akademik Nikolaj Strakhov of the Knipovich Ridge** (Updated since IBCAO v3 with higher resolution)^64^ http://atlantic.ginras.ru/download/exp/grd/grd_data.html
Geological Survey of Canada (GSC), Canadian Hydrographic Service (CHS)	**Multibeam and single beam bathymetry from CCGS Louis St-Laurent:** ***Single beam*** **LSL2007** ^65^ **LSL2008** ^66^ **LSL2009** ^67^ **LSL2010** ^68^ **LSSL2011** ^69^ ***Multibeam*** **LSSL2014** ^70^ **LSSL2015** ^71^ **LSSL2016** ^72^
Geological Survey of Denmark and Greenland (GEUS)	**Single beam data acquired during seismic exploration surveys of the Greenland continental margin provided by GEUS:** This contribution consists of >30 surveys carried out by various exploration companies for which the moratorium of the single beam bathymetry has expired. https://eng.geus.dk/
Geological Survey of Denmark and Greenland (GEUS), Danish Geodata Agency	**Multibeam bathymetry collected by Fugro for Demark’s extended continental shelf claim:** No publication available
Geological Survey of Denmark and Greenland (GEUS), Stockholm University and Swedish Polar Research Secretariat	**Multibeam bathymetry from Swedish icebreaker Oden acquired during the Lomonosov Ridge off Greenland (LOMROG) Expeditions 2007–2012 and East Greenland Ridge Expeditions (EAGER) 2011:** **LOMROG, 2007, Central Arctic Ocean** ^73,74^ **LOMROG 2009, Central Arctic Ocean** ^75^ **LOMROG 2012, Central Arctic Ocean** ^38,76^ **EAGER 2011, East Greenland Ridge** ^77^
Geological Survey of Sweden (SGU)	**Hoburg’s shoal survey from 2016/2017** ^78^ https://www.sgu.se/samhallsplanering/hav-och-kust/stod-till-havsplanering-och-forvaltning/projekt-hoburgs-bank/
GEOMAR Helmholtz Centre for Ocean Research Kiel	**Multibeam data from RV Maria S. Merian:** **05/03, 2007, Ilulissat Ice Fjord** ^79^ https://www.geomar.de/en/centre/
Global Multi-resolution Topography Data Synthesis (GMRT)	**GMRT version 3.5:** A multi-resolutional compilation of edited multibeam sonar data collected by scientists and institutions worldwide, that is reviewed, processed and gridded by the MGDS Team and merged into a single continuously updated compilation of global elevation data, provided at 15 arc sec resolution to GEBCO. https://www.gmrt.org/
Greenland Institute of Natural Resources (GINR)	**Crowd source data and multibeam data provided through Greenland Institute of Natural Resources**: These data include single beam soundings collected by GINR vessels Martek Aps, Kisaq, Greenland Police and Polar Seafood and multibeam bathymetry collected by Sanna in Nuup Kangerlua (Godthaabsfjord), Ameralik and Fyllas Bank of West Greenland in 2018. https://natur.gl/
IceBridge BedMachine Greenland	**IceBridge BedMachine Greenland, Version 3**: Greenland under-ice topography/bathymetry gridded compilation. Gridded resolution is 150 × 150 m on a Polar Stereographic projection^21^ http://nsidc.org/data/IDBMG4
International Hydrographic Organization Data Center for Digital Bathymetry (IHO DCDB)	**Bathymetric Soundings extracted from the data maintained by the International Hydrographic Organization (IHO) Data Center for Digital Bathymetry (DCDB) at the US National Centers for Environmental Information (NCEI):** https://www.ngdc.noaa.gov/iho/
Japan Agency for Marine-Earth Science and Technology (JAMSTEC)	**Multibeam bathymetry collected with Japanese RV Mirai extracted from Data and Sample Research System for Whole Cruise Information in JAMSTEC:** **MR00_K06:** http://www.godac.jamstec.go.jp/darwin/cruise/mirai/MR00-K06/e **MR02_K05:** http://www.godac.jamstec.go.jp/darwin/publication/mirai/mr02-k05/e **MR04_K05:** No publication available **MR99_K05:** http://www.godac.jamstec.go.jp/darwin/cruise/mirai/mr99-k05_leg1/e http://www.godac.jamstec.go.jp/darwin/e
Korean Polar Research Institute (KOPRI)	**Multibeam data from Korean RV Araon expeditions:** **ARA02B and ARA03B** ^80^ **ARA04C:** No publication available https://eng.kopri.re.kr
Maersk	Single beam navigation data from Baffin Bay seismic surveys: No publication available Navigation data from 2D-seismic surveys for exploration of hydrocarbons in Baffin Bay, West Greenland, in 2012, conducted by Polarcus DMCC for Maersk Oil. Released to Greenland Institute of Natural Resources for the purpose of preparation for publication in IBCAO/GEBCO.
MAREANO; Norwegian Hydrographic Service (NHS)	Bathymetric model of the Norwegian continental shelf compiled by the MAREANO project: This gridded bathymetric model (incorporated at a resolution of 50 × 50 m) has been produced by using high quality hydrographic survey data, primarily multibeam. In ocean areas, the coverage is largely dependent on the surveys organized by the MAREANO program (www.mareano.no/en). The coverage area is extended continuously and the data is updated whenever new hydrographic surveys are finished being processed. https://kartkatalog.geonorge.no/metadata/67a3a191–49cc-45bc-baf0-eaaf7c513549
Nansen Environmental and Remote Sensing Center	**Single beam from RH SABVABAA (Hoovercraft) drifts in the central Arctic Ocean:** **Drifts in 2011 and in 2014/2015** ^81^
NASA-Ocean Melting Greenland project, Caltech’s Jet Propulsion Laboratory and the University of California Irvine	**Multibeam bathymetry acquired by the Ocean Melting Greenland Project (OMG) 2013–2018 along the coast of Greenland from airborne marine gravity and ship-based observations** ^8,31,32,82–88^ https://omg.jpl.nasa.gov/portal/
National Geospatial-Intelligence Agency (NGA)	**Single beam data from Melville Bay, Greenland, contributed by NGA:** No metadata included on contribution
Lamont-Doherty Earth Observatory, Columbia University, Earth Institute (R/V Marcus G. Langeth expeditions)	**Multibeam bathymetry from R/V Marcus G. Langseth:** **MGL 1112, 2011, Chukchi Sea** ^89,90^ **MGL 1109, 2011, Gulf of Alaska** ^91^
Northeast Greenland Digital Bathymetric Model	Digital bathymetric model of Northeast Greenland (gridded compilation)^28^
Norwegian Hydrographic Service (NHS)	Svalbard bathymetry grid based on multibeam bathymetry: Released in 2016, this dataset includes modern multibeam data from surveys up until autumn 2015. Data is originally at 10 × 10 m, but down sampled to 100 × 100 m during the incorporation. https://www.kartverket.no/
The Norwegian Petroleum Directorate (NPD)	Multibeam bathymetry collected on behalf of the Norwegian Petroleum Directorate: The multibeam mapping was carried out by Gardline Ltd. https://www.gardline.com/
Norwegian Polar Institute (NPI)	Svalbard topography grid: New topographical data of Svalbard with updated glacial fronts from satellite imaging. https://toposvalbard.npolar.no/ http://www.npolar.no/no/
Olex AS, Norway	**Crowd source bathymetry provided by Olex:** These data are primarily single beam soundings collected by fishing vessels using the Olex acquisition system. The data are provided gridded at a resolution of 400 × 400 m. www.olex.no
Shell	**Single beam navigation data from Baffin Bay seismic surveys:** No publication available Navigation data from 2D-seismic surveys for exploration of hydrocarbons in Baffin Bay, West Greenland, in 2012, conducted by Polarcus DMCC for Royal Dutch Shell. Released to Greenland Institute of Natural Resources for the purpose of preparation for publication in IBCAO/GEBCO.
Swedish Polar Research Secretariat and Stockholm University	**Multibeam and single beam data from expeditions with Swedish icebreaker Oden:** The LOMROG and EAGER expeditions are listed separately above. ***Single beam*** **Arctic Ocean 1991, 1996, 2001** ^92–94^ ***Multibeam*** **AGAVE 2007** ^95^ **NEGC 2008**, Operated by Statoil A/S, no publication available **SAT 2008, 2009** ^96^ **SWERUS-C3 2014 Expedition** ^37,97,98^ **Petermann 2015 Expedition** ^30,99^ **Arctic Ocean 2016 Expedition** ^72^ **Ryder 2019 Expedition** Oden Mapping data: https://oden.geo.su.se/
Stockholm University, University of New Hampshire and Ola Skinnarmo	**Multibeam bathymetry acquired in Melville Bay, west Greenland, during the VEGA-Greenland Expedition 2013, with SY Explorer of Sweden** ^100^
TelePost Greenland A/S	**Greenland Connect Nord multibeam bathymetry from south-west Greenland:** No publication available Multibeam survey for offshore and inshore telecommunication cable from Nuuk to Aasiaat. Released to Greenland Institute of Natural Resources for the purpose of preparation for publication in IBCAO/GEBCO.
The University Centre in Svalbard (UNIS)	**Multibeam bathymetry from Svalbard, from seven cruises with RV Helmer Hanssen:** **JM09H** **JM10** ^101^ **HH11** ^102^ **HH12** ^103^ **HH13-NAL** ^104^ **HH13-SF** ^105^ **HH14**, No publication available https://www.unis.no/
University of Alaska Fairbanks and its College of Fisheries and Ocean Sciences	**Alaska Region Digital Elevation Model (ARDEM) Version 2.0** ^33^
University of Bremen, MARUM - Center for Marine Environmental Sciences	Multibeam data from western Svalbard region (Vestnesa Ridge) with MARUM RV Heincke^106^ HE449: https://www.marum.de/en/Research/RV-HEINCKE-HE449-1-August-22-August-2015-Trondheim-Tromso.html HE450: https://www.marum.de/en/Research/RV-HEINCKE-HE450-25-August-8-September-2015-Tromso-Tromso.html https://www.marum.de/en/index.html
University of New Brunswick, Ocean Mapping Group	**Multibeam bathymetry acquired with Canadian CCGS Amundsen:** Multibeam data from expeditions between 2003–2011 and 2013 are provided through the Ocean Mapping Group at University of New Brunswick separately from the NONNA-100 compilation where they also are included. http://www.omg.unb.ca/arctic-mapping/
University of New Hampshire, Center for Coastal and Ocean Mapping/Joint Hydrographic Center	**Multibeam bathymetry from U.S. Law of the Sea cruise to map the foot of the slope and 2500-m isobath of the US Arctic Ocean margin carried Center for Coastal and Ocean Mapping/Joint Hydrographic Center, University of New Hampshire**: https://ccom.unh.edu/theme/law-sea/arctic-ocean **HLY1603** ^107^ **HLY1202** ^108^ **HLY1102** ^109^ **HLY0905** ^110^ **HLY0805** ^111^ **HLY0703** ^112^ **HLY0302** ^113,114^ **HLY0405** ^115^ **HLY0503** ^116^ Bathymetry are in addition provided from the following expeditions with USCGC Healy through the Center for Coastal and Ocean Mapping/Joint Hydrographic Center, or retrieved from the IHO-DCDB: HLY0201, HLY0203, HLY0204, HLY0304, HLY0402, HLY0403, HLY0404, HLY0501, HLY0502, HLY0602, HLY0804, HLY0806, HLY0904, HLY1002
United States Geological Survey (USGS); National Geospatial-Intelligence Agency (NGA)	**Global Multi-resolution Terrain Elevation Data 2010 (GMTED2010)** https://www.usgs.gov/land-resources/eros/coastal-changes-and-impacts/gmted2010?qt-science_support_page_related_con=0#qt-science_support_page_related_con
US Navy	**Bathymetry from the Arctic region collected from US Navy nuclear submarines:** ***Single beam*** USS Topeka, 2012 USS New Hampshire, 2011 USS Connecticut, 2011 ***Single beam released in batches with no connection to specific submarine*** 1992–2000; 1985–1992; 1958–1985, 2001–2005 ***Single beam from the SCICEX program 1993–1998, and swath bathymetry from 1999*** SCICEX-93; USS Pargo SCICEX-95; USS Cavalla SCICEX-96; USS Pogy SCICEX-97; USS Archerfish SCICEX-98; USS Hawkbill SCICEX-99; USS Hawkbill (Swath bathymetry aquired with the SCAMP system, see main text)^39,117^
Woods Hole Oceanographic Institution (WHOI)	**Multibeam data from RV Knorr provided through WHOI Data Library and Archives:** **KN166-14, 2002, North Atlantic:** No publication found https://www.whoi.edu/
